# Concomitant Inhibition of IRE1α/XBP1 Axis of UPR and PARP: A Promising Therapeutic Approach against c-Myc and Gammaherpesvirus-Driven B-Cell Lymphomas

**DOI:** 10.3390/ijms23169113

**Published:** 2022-08-14

**Authors:** Rossella Benedetti, Andrea Arena, Maria Anele Romeo, Maria Saveria Gilardini Montani, Roberta Gonnella, Roberta Santarelli, Pankaj Trivedi, Mara Cirone

**Affiliations:** Department of Experimental Medicine, Sapienza University of Rome, Viale Regina Elena 324, 00161 Rome, Italy

**Keywords:** Burkitt lymphoma, UPR, IRE1α/XBP1, c-Myc, DDR, BRCA-1

## Abstract

It is emerging that targeting the adaptive functions of Unfolded Protein Response (UPR) may represent a promising anti-cancer therapeutic approach. This is particularly relevant for B-cell lymphomas, characterized by a high level of constitutive stress due to high c-Myc expression. In this study, we found that IRE1α/XBP1 axis inhibition exerted a stronger cytotoxic effect compared to the inhibition of the other two UPR sensors, namely PERK and ATF6, in Burkitt lymphoma (BL) cells, in correlation with c-Myc downregulation. Interestingly, such an effect was more evident in Epstein-Barr virus (EBV)-negative BL cells or those cells expressing type I latency compared to type III latency BL cells. The other interesting finding of this study was that the inhibition of IRE1α/XBP1 downregulated BRCA-1 and RAD51 and potentiated the cytotoxicity of PARP inhibitor AZD2661 against BL cells and also against Primary Effusion Lymphoma (PEL), another aggressive B-cell lymphoma driven by c-Myc and associated with gammaherpesvirus infection. These results suggest that combining the inhibition of UPR sensors, particularly IRE1α/XBP1 axis, and molecules involved in DDR, such as PARP, could offer a new therapeutic opportunity for treating aggressive B-cell lymphomas such as BL and PEL.

## 1. Introduction

Burkitt lymphoma (BL) is a malignant B-cell lymphoma whose pathogenesis is strictly linked to c-Myc-translocation/hyper-expression and, in its endemic form, also to EBV infection [1]. The virus is carried out in a latent state in these cells with different sets of latent viral gene expression. Type I latency is characterized by Epstein–Barr nuclear antigen (EBNA)1, *Epstein-Barr* encoding region (EBER) and BART expression, whereas type III latency includes a broader latent viral gene expression comprising of EBNA2, EBNA3A, -3B, -3C, the latent membrane proteins (LMPs) and BHRF1 [2]. Besides BL, c-Myc overexpression drives cell proliferation of a variety of solid and hematological cancers, including Diffuse Large B-Cell Lymphoma (DLBL) and Primary Effusion Lymphoma (PEL), although its translocations/mutations do not frequently occur in most of these cancers [3,4]. c-Myc overexpression promotes an increase of protein synthesis [5] and this effect may trigger ER stress, to which cancer cells attempt to adapt by activating the Unfolded Protein Response (UPR). This effect renders c-Myc-driven cancers particularly dependent on the activation of such a response [6]. Among the three UPR sensors, IRE1α, PERK, and ATF6, c-Myc has been reported to mainly activate the IRE1α/XBP1 axis [7], an effect that occurs also in BL [8]. Interestingly, XBP1s in turn may transactivate c-Myc, as reported for example in the case of prostate cancer [9]. In this regard, we have recently shown that targeting c-Myc reduces the constitutive activation of XBP1s, unbalancing UPR towards cell death. Moreover, c-Myc inhibition impaired DNA damage response (DDR) both in multiple myeloma (MM) and in PEL cells [10]. Indeed, XBP1s have the capacity to upregulate the expression of several molecules involved in DDR, either belonging to Homologous Repair (HR) and non-homologous end joining (NHEJ) repair. The cross-talk between UPR and DDR has been extensively reviewed [11] and we have recently contributed to dissect the relationship between these as well as the other adaptive responses that sustain the survival of cancer cells [12]. Based on this background, in this study, we investigated the impact of UPR inhibition on BL cell survival, focusing on IRE1α which is known to be the more important UPR sensor for the survival of a variety of hematological cancers [13]. At the molecular level, we analyzed the effect of UPR sensor inhibition on the expression level of c-Myc. We also evaluated the expression of BRCA-1 and RAD51, molecules involved in HR, an error-free DNA repairing pathway, which is essential for cancer cell integrity [14]. Finally, the possibility to potentiate the cytotoxicity induced by IRE1α/XBP1 axis inhibition by combining it with PARP inhibitors was evaluated. Indeed, it is known that defects in the HR pathway may render cancer cells more susceptible to treatment with PARP inhibitors [15], PARPs being mainly involved in other DNA repairing pathways such as NHEJ and BER, activated in response to single strand DNA brakes [16]. We finally extended this study to PEL cells, in which c-Myc is hyper-expressed, although not translocated, in which the IRE1α/XBP1 axis is known to sustain cell survival [17] and whose pathogenesis is linked to another oncovirus, Kaposi’s Sarcoma-Associated Herpesvirus (KSHV) and in many cases also to EBV. Whether the inhibition of IRE1α/XBP1 axis and PARP could be more cytotoxic than the single treatments against PEL was also evaluated, to assess if this therapeutic strategy could be widened against c-Myc-and gammaherpesvirus-driven B-cell lymphomas.

## 2. Results

### 2.1. IRE1α/XBP1 Axis Is the UPR Sensor Most Involved in Survival of BL Cells, Particularly in EBV-Negative and Type I Latency Cells

**Figure 1:**
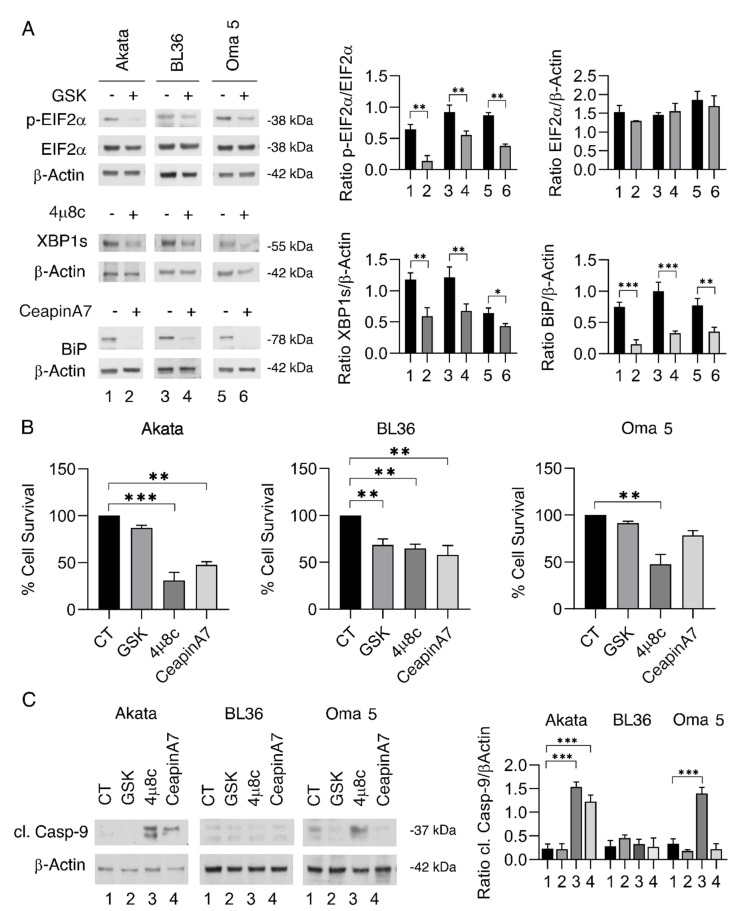
**Effects of PERK, IRE1-alpha, and ATF6 sensors inhibition in BL cells.** Akata, BL36, and Oma 5 cells were treated with GSK2606414 (GSK) (0.5 μM), 4μ8C (15 μM), or CeapinA7 (12 μM). The untreated cells were used as control (CT). (**A**) Protein expression levels of p-EIF2α, EIF2α, XBP1s, and BiP were evaluated by Western blot analysis. β-Actin was used as loading control. The histograms represent the densitometric analysis of the ratio of specific protein and the appropriate control of three different experiments. The data are shown as the mean plus S.D. (**B**) Cell viability was measured by a Trypan Blue exclusion assay, and the histograms represent the mean plus S.D. of live cells as percent of untreated control cells of three different experiments. (**C**) Protein expression level of cleaved Caspase-9 (cl. Casp-9) was evaluated by Western blot analysis. β-Actin was used as loading control. The histograms represent the densitometric analysis of the ratio of cl. Casp9/β-Actin of three different experiments. Data are represented as the mean plus S.D. *p* value: * *p* < 0.05; ** *p* < 0.001; *** *p* < 0.0001.

Three BL cell lines, namely Akata (type I latency), BL36 (Type III latency), and Oma 5 (EBV-negative) [18], were treated with specific inhibitors of the three branches of UPR, namely PERK, IRE1α/XBP1, and ATF6. Towards this aim, we used GSK2606414 (GSK), 4μ8C, and CeapinA7, drugs known to inhibit PERK, IRE1α/XBP1, and ATF6, and found that all of them efficiently inhibited their targets, p-EIF2α, XBP1s, and BIP, respectively (Figure 1A). The inhibition of all three UPR sensors affected BL cell survival (Figure 1B) while it did not affect that of primary B lymphocytes (Figure S1), according to the knowledge that normal cells are less dependent than cancer cells on UPR activation for their survival [12]. However, 4μ8C, the IRE1α/XBP1 inhibitor, was more efficient against Akata and Oma 5, as BL36 cells displayed sensitivity to 4μ8C similar to that induced by GSK and CeapinA7 (Figure 1B). Accordingly, the caspase 9 cleavage, which indicates the activation of an intrinsic apoptotic pathway, was observed in Oma 5 and Akata cells following the 4μ8C treatment and to a lesser extent by CeapinA7 in the latter cell line (Figure 1C). All together, these results suggest that the inhibition of IRE1α/XBP1 axis was more cytotoxic than the inhibition of the other two UPR sensors in EBV-negative Oma 5 BL and Akata expressing type I latency.

### 2.2. The Cytotoxic Effect of UPR Sensor Inhibition Correlates with the Downregulation of c-Myc 

**Figure 2:**
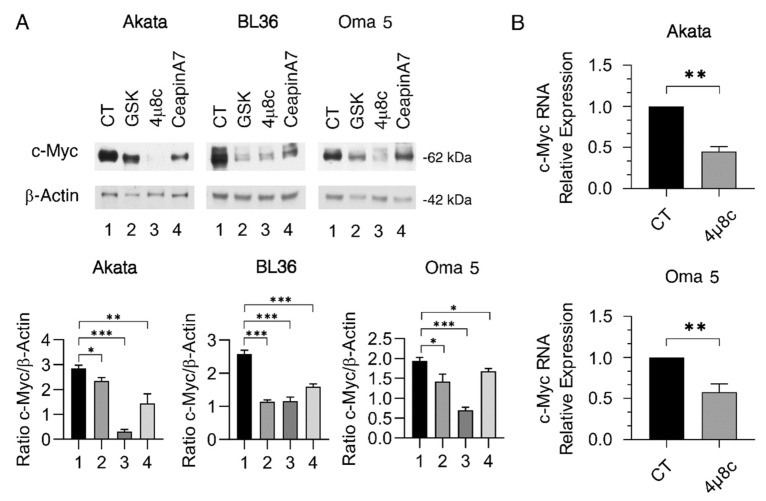
**UPR sensor inhibitors affect c-Myc expression**. Akata, BL36, and Oma 5 cells were treated with GSK2606414 (GSK) (0.5 μM), 4μ8C (15 μM), or CeapinA7 (12 μM) for 24 h. The untreated cells were used as control (CT). (**A**) c-Myc expression was evaluated by Western blot analysis. β-Actin was used as loading control and one representative experiment is shown. The histograms represent the densitometric analysis of the ratio of c-Myc/β-Actin of three different experiments. Data are represented as the mean plus S.D. (**B**) qRT-PCR of c-Myc in Akata and Oma 5 cells treated or not with 4μ8C (15 μM) for 24 h. The data are expressed relative to reference gene B2M. The histograms represent the mRNA expression levels of c-Myc genes of three different experiments. The data are represented as the mean relative to the control plus S.D. *p* value: * *p* < 0.05; ** *p* < 0.001; *** *p* < 0.0001.

Given the strict interplay between IRE1α/XBP1 and c-Myc, previously demonstrated by us and others [7,19], and the key role of c-Myc in BL growth/survival, we next investigated whether the higher cytotoxic effect of 4μ8C observed in Akata and Oma 5 could correlate with a stronger downregulation of c-Myc in these cells compared to BL36. As shown in Figure 2A, we found c-Myc expression level was more strongly reduced in Akata and Oma 5 than in BL36 in which 4μ8C downregulated c-Myc similarly to GSK and CeapinA7. To evaluate if the downregulation of c-Myc could occur at the transcriptional level, we performed q-RT-PCR and found the c-Myc mRNA was reduced by 4μ8C in both Akata and Oma 5 (Figure 2B), suggesting that c-Myc was transcriptionally modulated by 4μ8C in these BL cells.

### 2.3. IRE1α/XBP1 Inhibition Increases DNA Damage by Reducing the Expression of BRCA-1 and RAD51, Particularly in Akata and Oma 5 BL Cells

**Figure 3:**
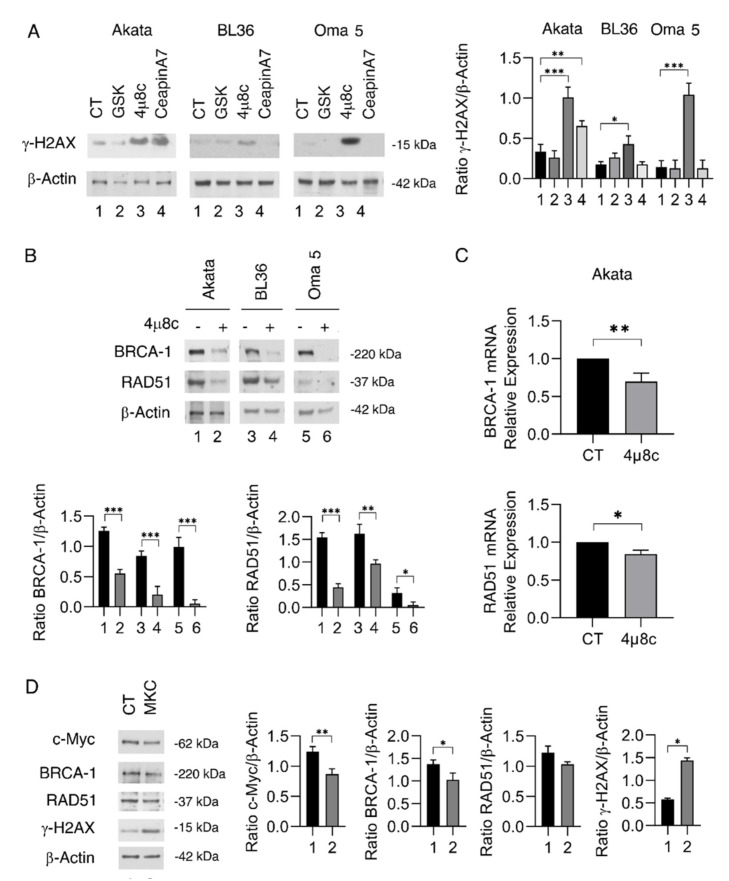
**IRE1-alpha inhibition by 4μ8C impairs DNA repair in BL cells**. Akata, BL36, and Oma 5 cells were treated with GSK2606414 (GSK) (0.5 μM), 4μ8C (15 μM), or CeapinA7 (12 μM) for 24 h. Untreated cells were used as control (CT). (**A**) Protein expression level of γ-H2AX was evaluated by Western blot analysis. β-Actin was used as loading control and one representative experiment is shown. The histograms represent the densitometric analysis of the ratio of γ-H2AX/β-Actin of three different experiments. Data are represented as the mean plus S.D. (**B**) Akata, BL36, Oma 5 cells were treated or not with 4μ8C (15 μM) for 24 h. Protein expression level of BRCA-1 and RAD51 was evaluated by Western blot analysis. β-Actin was used as loading control and one representative experiment is shown. The histograms represent the densitometric analysis of the ratio of specific protein and β-Actin of three different experiments. Data are represented as the mean plus S.D. (**C**) qRT-PCR of BRCA-1 and RAD51 in Akata cells treated or not with 4μ8C (15 μM) for 24 h. Data are expressed relative to reference gene B2M. The histograms represent the mRNA expression levels of BRCA-1 and RAD51 genes of three different experiments. Data are represented as the mean relative to the control plus S.D. (**D**) Akata cells were treated with MKC-8866 (MKC) (30 μM) for 24 h. Protein expression level of c-Myc, BRCA-1, RAD51, γ-H2AX was evaluated by Western blot analysis. β-Actin was used as loading control and one representative experiment is shown. The histograms represent the densitometric analysis of the ratio of specific protein and β-Actin of three different experiments. Data are represented as the mean plus S.D. *p* value: * *p* < 0.05; ** *p* < 0.001; *** *p* < 0.0001.

UPR and DDR are known to be strictly interconnected responses. In particular, XBP1s and its cross-talk with c-Myc can affect DDR [11]. Moreover, a critical role of PERK and ATF6 in DDR regulation has also been demonstrated [17,20,21]. Therefore, we evaluated whether the inhibition of PERK, IRE1α, and ATF6 could increase DNA damage in terms of H2AX phosphorylation. We found that 4μ8C was more effective compared to the other UPR sensor inhibitors to induce such an effect, particularly in Akata and Oma 5 cells (Figure 3A), in correlation with the higher cytotoxic effect observed in these cells. Of note, CeapinA7, which reduced cell survival in Akata, was also able to induce DNA damage in this cell line (Figure 3A). Taken together, these results suggest that cell survival, c-Myc expression, and DNA damage are strictly interconnected events in BL cells. We then evaluated whether defects of HR molecules could underly the strong DNA damage induced by IRE1α/XBP1 inhibition. As shown in Figure 3B, we found that BRCA-1 and RAD51 were downregulated by such treatment, the latter more strongly in Akata and Oma 5, and that it occurred also at mRNA level in these cells (Figure 3C). BRCA-1 and RAD51 are molecules essential to preserving cell integrity, and have been previously shown to be under XBP1s and c-Myc control [22]. We next evaluated the possibility of obtaining the effects induced by 4μ8C by using another IRE1α/XBP1 inhibitor, namely MKC8866, and found that it induced similar effects, although they were less efficient than 4μ8C (Figure 3D).

### 2.4. IRE1α/XBP1 Axis Inhibition Potentiates the Cytotoxic Effect of AZD2461 PARP Inhibitor 

**Figure 4:**
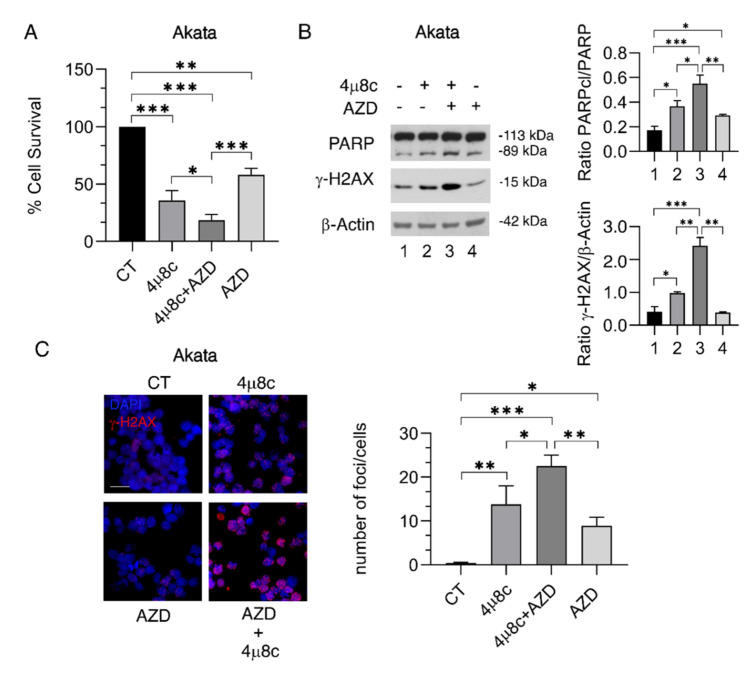
**Inhibition of IRE1-alpha/XBP1 axis increases the cytotoxic effect of PARP inhibition in BL cells.** Akata cells were pre-treated with 4μ8C (15 μM) for 1 h followed by treatment with AZD2461 (AZD) (40 μM). (**A**) After 24 h, the cell viability was measured by a Trypan Blue exclusion assay; the histograms represent the mean plus S.D. of live cells as percent of untreated control cells. (**B**) Protein expression level of PARP and γ-H2AX was evaluated by Western blot analysis. β-Actin was used as loading control and one representative experiment of three is shown. The histograms represent the densitometric analysis of the ratio of specific protein and the appropriate control of three different experiments. The data are represented as the mean plus S.D. (**C**) γ-H2AX foci (red) were assessed by IFA in Akata cell line. DAPI (blue) was used for nuclear staining. One representative experiment out of three is reported. The histograms represent the mean plus S.D. of the number of foci/cell obtained by three different experiments. Bars = 20 μm. *p* value: * *p* < 0.05; ** *p* < 0.001; *** *p* < 0.0001.

Cancers carrying defects in BRCA-1 are known to be more susceptible to the cytotoxic effect of PARP inhibitors [22], and therapeutic strategies able to reduce the expression of BRCA-1 as well as other HR molecules, such as RAD51 [23], or to exacerbate DNA damage [24] can be used to potentiate the cytotoxicity of PARP inhibitors. Therefore, in this study the combination of 4μ8C and AZD2461 PARP inhibitor was evaluated against Akata cells in which BRCA-1 and RAD51 were efficiently downregulated by 4μ8C. As shown in Figure 4A,B, such a drug combination reduced Akata cell survival and induced a stronger PARP cleavage compared to single treatments. As expected, the AZD2461/4μ8C combination also triggered stronger DNA damage in these cells, which was evidenced by the increased phosphorylation of H2AX (Figure 4B) and by the high number of H2AX-positive foci (Figure 4C).

### 2.5. 4μ8C Induces in PEL Cells Effects Similar to Those Observed against BL Cells 

**Figure 5:**
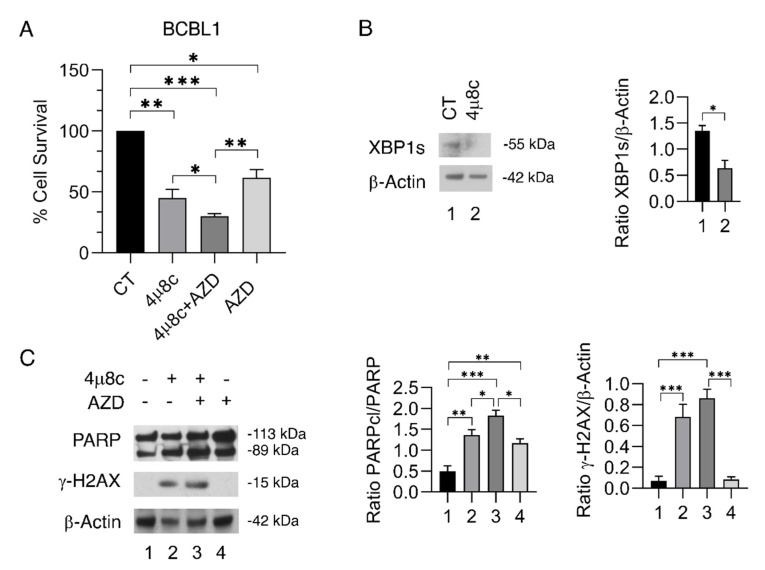
**The inhibition of IRE1-alpha/XBP1 axis increases the cytotoxic effect of PARP inhibition also in PEL cells.** BCBL1 cells were pre-treated with 4μ8C (40 μM) for 1 h and subsequently treated with AZD2461 (AZD) (40 μM). (**A**) After 24 h, the cell viability was measured by a Trypan Blue exclusion assay. The histograms represent the mean plus S.D. of live cells as percent of untreated control cells. (**B**, **C**) Protein expression level of XBP1s, PARP and γ-H2AX was evaluated by Western blot analysis. β-Actin was used as loading control and one representative experiment is shown. The histograms represent the densitometric analysis of the ratio of specific protein and β-Actin of three different experiments. Data are represented as the mean plus S.D. *p* value: * *p* < 0.05; ** *p* < 0.001; *** *p* < 0.0001.

We then extended this study to BCBL1 PEL cells, a B-cell lymphoma in which the pro-survival role of XBP1s [25] and the cross-talk of this molecule with c-Myc has been previously shown [10]. Interestingly, 4μ8C, which efficiently reduced XBP1s expression (Figure 5B), increased the cytotoxicity of AZD2461 also in these cells compared to the single treatments (Figure 5A). Again, in these cells, such a combination induced a stronger PARP cleavage (Figure 5C) and DNA damage (Figure 5C) in comparison to 4μ8C and AZD2461 alone. These results suggest that the combination of inhibitors of IRE1α and PARP could be a promising therapeutic strategy against lymphomas other than BL, given that it was effective also against PEL cells that are known to respond poorly to therapy [26].

## 3. Discussion

c-Myc overexpression is strongly involved in cell proliferation and pathogenesis of BL [27,28]. This B-cell lymphoma also often carries EBV infection with a different pattern of viral protein expression, which may influence the response to therapy [29]. It has been reported that IRE1α/XBP1 signaling is particularly important for growth/survival of Myc-overexpressing BL cells, dependent on elevated stearoyl-CoA-desaturase 1 (SCD1) activity, and that the inhibition of IRE1α/XBP1 could potentiate the cytotoxicity of chemotherapies already used against BL [8]. In this study, we observed that IRE1α/XBP1 inhibition by 4μ8C was more effective in reducing BL cell survival compared to the inhibition of the other two UPR sensors, although such a difference was more evident in type I latency expressing Akata and EBV negative Oma 5 cells in comparison to type III latency BL36 cells. Interestingly, the presence or the absence of EBV can also influence the susceptibility to superinfection by other herpesviruses [30]. Identifying the differences between lymphoma cells harboring or not harboring viral infection or expressing a different pattern of viral antigens can help to design more appropriate therapies. In the case of EBV-associated lymphomas, our results suggest that for BL, which mainly express type I latency, the targeting of IRE1α/XBP1 may be more promising than for post-transplant EBV-positive lymphomas, mainly characterized by type III latency. However, as for other cancers, lymphoma cells overexpressing c-Myc are characterized by a high level of constitutive stress, which renders them highly dependent on the activation of UPR sensors such as IRE1α [13]. The cytotoxic effect of IRE1α/XBP1 inhibition by 4μ8C correlated with the downregulation of c-Myc in BL cells, given that the latter may engage a positive feedback loop with XBP1s to sustain the survival of cancers [6,10]. Targeting UPR has also been reported to be effective in reducing cell survival of mutp53-carrying cancers through the upregulation of the pro-apoptotic UPR molecule CHOP [31], even if the presence of mutp53 may render cancer cells more resistant to such treatment [32]. However, in a recent study, we found that the inhibition of the IRE1α/XBP1 axis efficiently reduced the survival of mutp53-carrying Multiple Myeloma (MM) cells [19], encouraging the use of this therapeutic strategy against mutp53-carrying hematological cancers also. In concordance with previous studies showing an interconnection between UPR and DDR [11], in this study we found that XBP1s inhibition resulted in a reduced expression of RAD51 and BRCA-1, both at protein and mRNA levels. This led to an impairment of DDR, in particular of the HR pathway, and to an increase of DNA damage in BL cells. Another interesting finding of this study is that, through this mechanism, 4μ8C sensitized BL cells to the cytotoxic effect of AZD2461 PARP inhibitor, identifying a new therapeutic combination that could be used against this aggressive B-cell lymphoma. Indeed, when drugs directly targeting DDR are used in combination with PARP inhibitors, they not only induce a stronger cytotoxic effect but may also help to reduce the resistance to PARP inhibitors that cancer cells often develop [16,33]. In particular, strategies targeting the HR may offer this opportunity [34], as PARPs, although contributors to all DNA repairing systems, are particularly involved in base excision repair (BER) and NHEJ rather than HR [35]. Interestingly, an impairment of HR can be induced by 4μ8C, as this drug, besides inhibiting a specific UPR sensor, can indirectly impair DDR. Of note, we found that the 4μ8C/AZD2461 combination was effective also against PEL cells, another B-cell lymphoma known to be strongly dependent on UPR activation, particularly on the IRE1α/XBP1 axis [25]. As for BL, PEL proliferation is driven by c-Myc together with the presence of the oncovirus KSHV and, in some cases, also of EBV [36]. The findings of this study suggest that this previously unexplored combinatorial therapeutic approach could have wider applications for the treatment of aggressive B-cell lymphomas such as those associated with gammaherpesviruses, even if more cell lines and in vivo experiments will be important to further support our results. The combination therapy tested in the present study could be also added to the evolving therapeutic landscape of aggressive B-cell lymphoma recently reviewed by Patriarca et al. [37].

## 4. Materials and Methods

### 4.1. Cell Cultures and Treatments 

BL cell line Akata established from a Japanese patient with Burkitt’s lymphoma was kindly provided by Prof. Takada [38], BL-36 cells were obtained from Prof. Klein laboratories [39], Oma 5 cells were isolated as EBV-negative clone from Oma-BL1 [18], and PEL cell line BCBL1 was kindly provided by Prof. P. Monini (National AIDS Center, Istituto Superiore di Sanità, Rome, Italy). Cells were maintained in RPM1-1640 (PAN-Biotech, Aidenbach, Germany) supplemented with 10% Fetal Bovine Serum (FBS) (Corning, Corning, NY, USA), 1% L-glutamine (100 μg/mL) (Aurogene, Rome, Italy), 1% streptomycin and penicillin (100 U/mL) (Aurogene, Rome, Italy) at 37 °C in a 5% CO^2^ incubator. 

Cells were plated in 6-well plates at a density of 3 × 10^5^ cells/mL in 2 mL and treated with 0.5 μM GSK2606414 (GSK) (PERK inhibitor) (S7307; Selleckem, Houston, TX, USA), 15 μM 4μ8C (IRE1 RNAse inhibitor) (SML0949; Sigma-Aldrich, Burlington, MA, USA), 12 μM CeapinA7 (ATF6a signaling blocker) (SML2330; Sigma-Aldrich, Burlington, MA, USA and 30 μM MKC-8866 (IRE1 RNase inhibitor) (HY-104040; MedChemExpress, Monmouth Junction, NJ, USA) for 24 h. The concentrations of the drugs were designed based on preliminary experiments and previous studies [25]. In some experiments, cells were plated in 6-well plates as reported above and were pre-treated 15 μM 4μ8C for 1 h, subsequently treated with 40 μM AZD2461 (PARP inhibitor) (SML1858; Sigma-Aldrich, Burlington, MA, USA) and cultured for additional 24 h. The untreated cells were used as control.

To evaluate the cytotoxic effects of these drugs on primary B lymphocytes, human peripheral blood mononuclear cells (PBMCs) from healthy donors were isolated by lymphocyte cell separation medium (CL5020; Cedarlane, Burlington, Canada) [40] and B lymphocytes were separated by immunomagnetic cell separation kit using anti-CD19-conjugated microbeads, according to the manufacturer’s instructions (130-050-301, Miltenyi Biotec, Bergisch Gladbach, Germany). B cells were cultured in RPMI-1640 complete medium, in 5% CO^2^-saturated humidity at 37 °C in 24-well plates at density of 2 × 10^6^ cells/mL and then treated with 0.5 μM GSK2606414, 15 μM 4μ8C or 12 μM CeapinA7 for 24 h. The untreated cells were used as control.

### 4.2. Cell Viability

The cell viability was evaluated by a Trypan Blue (Sigma-Aldrich, Burlington, MA, USA) exclusion assay after 24 h of treatments. The cells were counted by light microscopy (Labovert FS Inverted Microscope, Leica Microsystems, Wetzlar, Germany) using a Neubauer hemocytometer. The experiments were performed in triplicate and repeated at last three times. 

### 4.3. Western Blot Analysis

After treatments, the cells were washed in 1X PBS (Aurogene, Rome, Italy), lysed in RIPA buffer (150 mM NaCl, 1% NP-40 (Calbiochem, San Diego, CA, USA), 50 mM Tris-HCl (pH 8), 0.5% deoxycholic acid (Sigma-Aldrich, Burlington, MA, USA), 0.1% SDS (Sigma-Aldrich, Burlington, MA, USA), protease and phosphatase inhibitors (Roche, Basel, Switzerland), and centrifuged at 14,000 rpm for 20 min at 4 °C by Sigma 1-15PK refrigerated centrifuge (Sigma Laborzentrifugen, Osterode am Harz, Germany). The protein concentration was measured by using the Bio-Rad Protein Assay (BIO-RAD laboratories GmbH, Munich, Germany), and 12 µg of protein was subjected to electrophoresis on 4–12% NuPAGE Bis-Tris gels (Life Technologies, UK) according to the manufacturer’s instruction. The gels were transferred to nitrocellulose membranes (Bio-Rad, Hercules, CA, USA) for 1 h in Tris-glycine buffer and the membranes were blocked in 1X PBS–0.1% Tween 20 (Serva, Heidelberg, Germany) solution containing 3% of BSA (Serva, Heidelberg, Germany), probed with specific antibodies and developed using ECL Blotting Substrate (Advansta, CA, USA).

### 4.4. Antibodies

To evaluate protein expression the following primary antibodies were used: rabbit polyclonal anti-phospho-eIF2α (Ser51) (9721; Cell Signaling, Danvers, MA, USA), rabbit polyclonal anti-eIF2α (9722; Cell Signaling, Danvers, MA, USA), rabbit polyclonal anti-XBP1s (24868-1-AP; Proteintech, Rosemont, IL, USA), anti-BiP/GRIP78 (11587-1-AP; Proteintech), rabbit polyclonal anti-Caspase 9 (10380-1-AP; Proteintech, Rosemont, IL, USA), rabbit polyclonal anti-c-Myc (10828-1-AP; Proteintech, Rosemont, IL, USA), mouse monoclonal anti-γ-H2AX (Ser 139) (sc-517348; Santa Cruz Biotechnology, Dallas, TX, USA), mouse monoclonal anti-BRCA-1 (OP92; EMD Millipore, Burlington, MA, USA), mouse monoclonal anti-RAD51 (G-9) (sc-377467; Santa Cruz Biotechnology, Dallas, TX, USA), and rabbit monoclonal anti-PARP (46D11) (9532; Cell Signaling, Danvers, MA, USA). Mouse monoclonal anti-β-actin (A5316; Sigma-Aldrich, Burlington, MA, USA) was used as loading control. The goat anti-Mouse IgGP Peroxidase Conjugate (401215; Sigma-Aldrich, Burlington, MA, USA) and the goat anti-Rabbit IgG Peroxidase Conjugate (DC03L; Sigma-Aldrich, Burlington, MA, USA) were used as secondary antibodies. 

### 4.5. Indirect Immunofluorescence Assay 

Indirect immunofluorescence assay (IFA) was performed to evaluate γ-H2AX foci formation. Akata cells were plated in 6-well plates as reported above, and were pre-treated with IRE1 RNAse inhibitor 4μ8C (15 μM) for 1 h, then treated with AZD2461 (10 μM), which is a PARP inhibitor, for an additional 24 h. After treatments, cells were washed with PBS, applied onto multi-spot microscope slides, and air-dried. Slides were then incubated with 2% paraformaldehyde (Electron Microscopy Science) for 30 min and permeabilized with 0.1% Triton X-100 (Sigma-Aldrich, Burlington, MA, USA) for 5 min. After three washes, cells were incubated with 1% glycine (Serva, Heidelberg, Germany), 3% BSA for a further 30 min. Thereafter, cells were incubated with mouse primary monoclonal antibody anti-γ-H2AX (phosphor-Ser 139) (sc-517; Santa Cruz Biotechnology, Dallas, TX, USA) for 1 h at room temperature. Subsequently, cells were incubated with a polyclonal conjugated-Cy3 sheep anti-mouse antibody (Jackson ImmunoResearch, Cambridge, UK) for 30 min at room temperature and stained with DAPI (Sigma-Aldrich) for 1 min at room temperature. Slides were analyzed with Apotome Axio Observer Z1 inverted microscope (Zeis, Wetzlar, Germany) equipped with an AxioCam MRM Rev.3 at 40× magnification. Foci number analysis was performed by Image J software (1.47 version, NIH, Bethesda, MD, USA).

### 4.6. RNA Isolation and Quantitative Real Time Polymerase Chain Reaction (qRT-PCR)

After treatments, total RNA from Akata and Oma 5 cells was isolated as described previously [41]. Briefly, total RNA was isolated with TRIzol™ Reagent (Invitrogen, Life Technologies Corporation, Carlsbad, CA, USA) according to the manufacturer’s instructions. The concentration and purity of RNA were determined at 260/280 nm using a Nanodrop (MaestroNano Micro-Volume Spectrophotometer, MaestroGen, Hsinchu, Taiwan). c-Myc, BRCA-1, and RAD51mRNA expression levels were analyzed using TaqMan gene expression assays (Applied Biosystems, Vilniaus, Lithuania): 2 µg of total RNA was reverse-transcribed into cDNA using High-capacity cDNA Reverse Transcription Kit (Thermo Fisher Scientific, Waltham, MA, USA) and a mastermix containing 2 µL cDNA (20 ng), 1 µL of TaqMan gene expression assays specific for c-Myc, BRCA-1, RAD51 (HS00153408-m1, HS01556193-m1, HS01556193-m1; Applied Biosystem, Vilniaus, Lithuania) and 10 µL of 2x TaqMan Fast Advance Master Mix was prepared for each PCR. The PCRs were run on an Applied Biosystem Real-Time thermocycler (Applied Biosystems, Vilniaus, Lithuania). Each amplification was performed in triplicate, and the average of three threshold cycles was used to calculate transcript abundance. The starting concentration of each specific product was divided by the starting concentration of reference gene B2M (HS99999907-m1; Applied Biosystem, Vilniaus, Lithuania) and this ratio was compared between treated/control groups.

### 4.7. Densitometry Analysis

The quantification of protein bands was performed by densitometric analysis using the Image J software (1.47 version, NIH, Bethesda, MD, USA).

### 4.8. Statistical Analysis

The results are represented as the mean plus standard deviation (SD) of at least three independent experiments, and a two-tailed Student’s t-test was used to demonstrate statistical significance. Difference was considered as statistically significant when *p*-value was at least < 0.05.

## Data Availability

The datasets generated and/or analyzed during the current study are available from the corresponding author upon reasonable request.

## Supplementary Materials

The following supporting information can be downloaded at: https://www.mdpi.com/article/10.3390/ijms23169113/s1.
