# Supplementary material for: Concomitant Inhibition of IRE1α/XBP1 Axis of UPR and PARP: A Promising Therapeutic Approach against c-Myc and Gammaherpesvirus-Driven B-Cell Lymphomas
Source: Int J Mol Sci. 2022 Aug 14;23(16):9113. doi: 10.3390/ijms23169113 (PMC9409055; doi:10.3390/ijms23169113)
Supplement: Supplementary file 1 [file ijms-23-09113-s001.zip › ijms-1841106-supplementary.pdf]

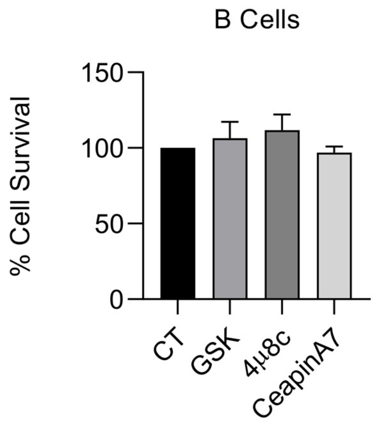

Figure S1. Primary B lymphocytes isolated from healthy donors were treated with UPR sensor inhibitors for 24 hours and viability was evaluated by trypan blue exclusion assay.
